# A novel approach utilizing Β-tricalcium phosphate/gelatin composite scaffolds incorporated with gentamycin- loaded chitosan microspheres for periodontal regeneration in two-wall intra bony defects

**DOI:** 10.1016/j.jobcr.2025.10.004

**Published:** 2025-10-15

**Authors:** Mohamed Hamdy Helal, Aya Anwar Alsherif, Yasmin Hamdy, Malak Yousef Mohamed Shoukheba, Khaled M. Ali, Mohamed Yehia Abdelfattah, Moustafa Nabil Aboushelib

**Affiliations:** a Oral Medicine, Periodontology, Oral Diagnosis, and Radiology Department, Faculty of Dentistry, Tanta University, Egypt; b Oral Biology Department, Faculty of Dentistry, Tanta University, Egypt; c Department of Surgery, Faculty of Veterinary Medicine, Cairo University, Giza, Egypt; d Oral Biology Department, Faculty of Dentistry, Beni-Suef University, Egypt; e Department of Basic Dental Sciences, Faculty of Dentistry, Ibn Sina University for Medical Sciences, Jordan; f Dental Biomaterials Department, Faculty of Dentistry, Alexandria University, Egypt

**Keywords:** Dentistry, Periodontal regeneration, Scaffolds, Biomaterials, Bony defects, Dogs

## Abstract

**Objectives:**

There is an increasing demand for innovative biomaterials, scaffolds and techniques in dental practices to improve regenerative therapies. Researchers studied periodontium regeneration using biologically compatible scaffolds to repair periodontal and bony defects. The present study aimed to evaluate the effect of β-tricalcium phosphate/gelatin composite scaffolds incorporated with gentamycin-loaded chitosan microspheres for periodontal regeneration in two-wall intra bony defects in Dogs.

**Design:**

12 bilateral intrabony defects (total 24 defects), 5 mm height, and 3 mm depth, were surgically created on the mesial surface of second mandibular premolars and allowed to become chronically healed in 12 mongrel male dogs. Dogs were randomly divided into three groups: Group I (8 defects) received β-tricalcium phosphate/gelatin composite scaffolds incorporated with gentamycin-loaded chitosan microspheres. Group II (8 defects) received the same scaffolds without gentamycin. Group III (8 defects) received open flap debridement (OFD) as a control. The animals were sacrificed at 8 weeks post-operatively for histological and histomorphometry analysis (n = 8, α = 0.05).

**Results:**

In comparison to the control, both test groups exhibited the highest percentage of newly formed bone height, newly formed bone area, newly formed cementum, and newly formed periodontal tissues, indicating a substantial (P < 0.001) increase in new regenerative tissue. However, in the control group, epithelial downgrowth predominated.

**Conclusions:**

Within the limitations of this study, the novel treatment approach using β-TCP/Gelatin Scaffolds with Gentamicin-Loaded Chitosan improved periodontal regeneration and wound healing in two-wall intra bony defects in Dogs.

## 1 Introduction

There is an increasing demand for innovative biomaterials, scaffolds and techniques in dental practices to improve regenerative therapies. Researchers studied periodontium regeneration using biologically compatible scaffolds to repair periodontal and bony defects. The present study aimed to evaluate the effect of β-tricalcium phosphate/gelatin composite scaffolds incorporated with gentamycin-loaded chitosan microspheres for periodontal regeneration in two-wall intra bony defects in Dogs. Periodontal disease is an inflammatory condition brought on by periodontopathogenic bacteria that, when the inflammation worsens, eventually destroys alveolar bone.^1^ In these circumstances, spontaneous regeneration typically does not take place, hence the treatment of periodontitis is centered on precautionary measures like the control of plaque and anti-inflammatory therapies.^2^ Tissue regeneration and reconstruction therapies, on the other hand, have attracted a lot of interest during the past few decades.^3^ Autogenous bone grafting is the gold standard for treating alveolar bone loss, but it has drawbacks like the need for a second surgery at the donor site, limiting bone availability.^4^ These issues can be avoided by placing artificial scaffold material at the site of the bone deficiency, which encourages osteogenesis in both quantity and quality.^5^^,^^6^ Porous scaffolds are common because they are essential for cell adhesion, proliferation, and differentiation in tissue engineering.^7^ Additionally, the substrate material's tendency for osseointegration is considerably increased by the presence of a rough surface.^8^ A broad-spectrum aminoglycoside antibiotic called gentamycin (GM) has a potent inhibitory impact on both gram-positive and gram-negative bacteria.^9^ Microspheres (MSs) are microscale, adaptable platforms used as medication carriers to improve dispersion and site-specific targeted delivery of medications, minimizing side effects and enhancing therapeutic impact.^10^^,^^11^ A popular choice for the manufacture of MS is chitosan (CS). CS is a biocompatible, all-natural polysaccharide that is also reasonably priced.^12^ In addition, CS has pharmacological properties like bactericidal and antibacterial activity.^13^ Infected bone has been successfully treated using scaffolds made of B-tricalcium phosphate/gelatin composite and gentamycin-loaded chitosan microspheres with consideration of potential effect of gentamicin on S. aureus intracellular proliferation and survival.^14^^,^^15^

Shirakata et al., 2022 published a study comparing periodontal regeneration in two groups, one of them by using recombinant human fibroblast growth factor‐2 (rhFGF-2) combined with β‐tricalcium phosphate, carbonate apatite while the other group utilized deproteinized bovine bone mineral (DBBM) in a canine one‐wall intra‐bony defect model and concluded that rhFGF-2/DBBM treatment promotes favorable periodontal regeneration.^16^ Due to the increased demand for aesthetic procedures in dental field, there is an increased interest in periodontal plastic surgery which result in the development of new surgical techniques and concepts for periodontal regeneration and treating bony defects in the periodontium using concentrated growth factor membrane^17^ and accompanied periodontal diseases as gingival recession using xenogenic collagen matrix instead of autologous connective tissue graft^18^

To the best of our knowledge there is no study was conducted on its effect on periodontal regeneration in two-wall intrabony defects, so our study was conducted to evaluate its effect in periodontal regeneration in a canine model.

The null hypothesis states that β-tricalcium phosphate/gelatin composite scaffolds combined with or without gentamycin-loaded chitosan microspheres are superior than open flap debridement in promoting periodontal regeneration in dogs.

## 2 Materials and methods

This research was carried out in the Department of Surgery, Anesthesiology, and Radiology Faculty of Veterinary Medicine at Cairo University. The Study protocol was approved by the ethics committee of Tanta University specifying conditions and constraints for conducting and publishing studies involving animal models (approval no #R-OMPDR-8-23-5). and followed the ARRIVE guideline.^19^ Scaffold Preparation β-tricalcium phosphate/gelatin composite scaffolds incorporated with gentamycin-loaded chitosan microspheres were prepared using the method of Liu et al., 2022.^14^

The sample size was calculated using G Power version 3.1.9.2.^20^ The minimal sample size was calculated based on a previous study aimed to examined the effects, on bioactivity, of a nano-β-TCP/collagen scaffold loaded with FGF-2, particularly on periodontal tissue wound healing. 22 they suggested that the β-TCP nanoparticle coating strongly improved the collagen scaffold bioactivity. Nano-β-TCP scaffolds containing FGF-2 are anticipated for use in periodontal tissue regeneration in one-wall infrabony defects of dogs. The sample size was calculated to assess the difference in newly formed bone area (primary outcome) among the three groups. Based on Ogawa et al. (2016) results to detect a standardized effect size of 0.518 in the newly formed bone area, with a power of 80 % and a significance level of 5 %, the minimum required sample size was found to be 8 specimens per group with a Total sample size equal to 24 specimens for the three treated groups.^21^ Any specimen loss from the study sample due to processing error was replaced to maintain the sample size.

### 2.1 Scaffold Preparation

Chitosan microspheres (CM) loaded with Gentamycin (Gm) were fabricated using the microcapsulation method after stirring and dissolving 10 g of chitosan and 5 g of Gentamycin in 500 mL of 1 % acetic acid (Millipore Merck, Germany) solution. 1.8 gm of CMs (GM) and 1.1 gm of Nano β-TCP (less than 100 nm, spheroidal shape, Nano Gate Company, Egypt) were added to 5 mL of 18 % gelatin (Loba – Chemie, India) solution at 37 °C. 150 mL of tripolyphosphate (TPP) (0.2 % w/v) was added in the dropwise method to precipitate the loaded microspheres.^22^ Final precipitation was assisted using a sodium hydroxide solution. After constant stirring at 5000 rpm for 5 min, 1.5 mL of 1 % genipin solution was added and stirred for another 30 min. The mixture was poured into prefabricated casts of the defect size and freeze-dried overnight. The casts allowed the fabrication of scaffolds that precisely fit the defect size.^19^

All scaffold specimens were sterilized using gamma radiation (the Egyptian Atomic Energy Authority).

### 2.2 Experimental animals and their housing protocol

Twelve mongrel dogs, weighing 12–14 kg, were chosen for the study and were kept at an animal healthcare facility in the Faculty of Veterinary Medicine at Cairo University. The dogs were in good general health. They were provided with a balanced diet of milk, broth, and meat during the study period. All animals were kept in individual stainless-steel cages with direct access to water, proper ventilation, as well as a 12-h light/dark cycles. The dogs were given a subcutaneous injection of atropine (0.05 mg/kg; Kwang Myung Pharmaceutical) to induce anesthesia. After 10 min of premedication, anesthesia was induced by injecting a mixture of 2 mg/kg Xylazine (Xyla-Ject; Adwia Pharmaceuticals) and 5.5 mg/kg ketamine hydrochloride (KETAMAX-50; Troikaa Pharma) into the cephalic vein of the forelimb and have been maintained with inhalation anesthesia. As well as a local infiltration (2 % with 1:100,000 epinephrine, Art pharms, Egypt).

### 2.3 Surgical protocol

The dogs were given a subcutaneous injection of atropine (0.05 mg/kg; Kwang Myung Pharmaceutical) to induce anesthesia. After 10 min of premedication, anesthesia was induced by injecting a mixture of 2 mg/kg Xylazine (Xyla-Ject; Adwia Pharmaceuticals, Egypt) and 5.5 mg/kg ketamine hydrochloride (KETAMAX-50; Troikaa Pharma, India) into the cephalic vein of the forelimb and have been maintained with inhalation anesthesia. As well as a local infiltration (2 % with 1:100,000 epinephrine, Art pharm, Egypt). Two-wall intrabony defects were surgically created bilaterally mesially to the mandibular second pre-molars in beagle dogs as follow.

**Fig. 1:**
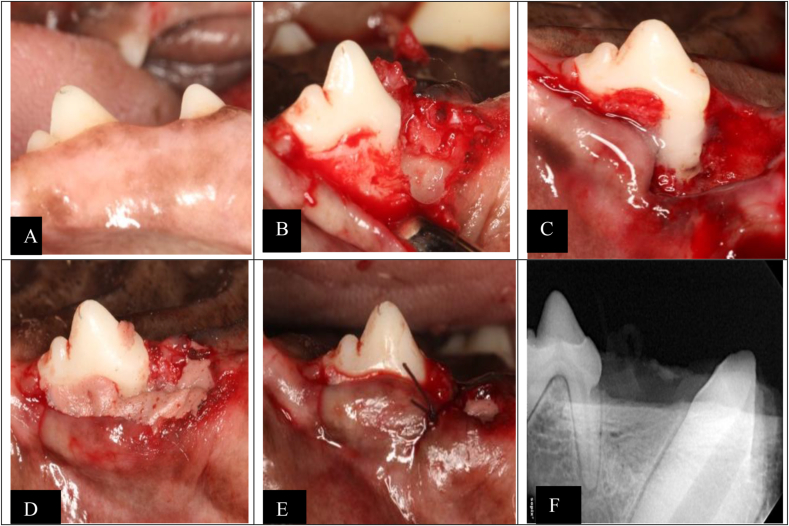
Photomicrographs illustrating the clinical procedures in the test group. (A): Pre-surgical, (B): Elevated Mucoperiosteal flap (C):chronic two-wall defect (D): Second reentry and removal of rubber base material (E): After insertion of a trimmed β-TCP/Gelatin scaffold with Gentamicin-Loaded Chitosan, wound closure and flap stabilization by interrupted suture, (F) Post-operative X-ray after 4 weeks.

The first pre-molars of both mandibular quadrants were extracted, and the sites were allowed to heal for 12 weeks. The remaining dentition received oral prophylaxis after the extraction procedure. In a second phase, the animals were anesthetized like in the first phase. The surgeries were performed by two well-trained periodontists with extensive experience in regenerative periodontal surgery (Y.H and M.H). Mucoperiosteal flaps were elevated, and a chronic “box-type” 2-wall intrabony defects of approximately 5 × 5 × 5 mm (mm) were surgically created by leaving the palatal bone wall intact (Fig. 1). Before bone removal, a coronal reference notch (notch A) was created with a round bur (diameter 1 mm) into the root at the initial alveolar crest. The defects were created at the mesial aspects of the second pre-molars of both mandibular quadrants by means of rotating and hand instruments. Subsequently, the roots were thoroughly scaled to remove the root cementum and PDL. Following root planning, a reference notch (notch B) was created at the apical extension of the defect in the same manner as the coronal notch. The notches served as reference points for the histomorphometric measurements. Thus, all periodontal tissues that would form coronally to the notch are newly formed periodontal tissue. Next, silicone impression material has been used to cover the defects and the assembly had been allowed to rest for one month. The treatment was performed according to the allocated group procedure. 24 defect sites per quadrant were randomly divided into 8 defects control and two test sites 8 defects each. After root biomodification using EDTA 24 % (PrefGel; Straumann) at the test site, trimmed CMs (GM)-β-TCP/gelatin composite scaffolds with and without gentamycin, in the shape of the defect, were softly compressed into the bony pocket (Fig. 1), whereas the control site was left empty. At the level of the bony defect, the flap was stabilized by means of a simple interrupted suture. After the surgeries, pain was controlled with morphine (0.3 mg/kg/IM/6 h) for 24 h and meloxicam (0.1 mg/kg/s.i.d/P.O.; Metacam, Boehringer Ingelheim, Barcelona, Spain) for 4 days. Antibiotics (amoxicillin 22 mg/kg/s.i.d./SC; Amoxoil retard, Syva, Leon, Spain) were administrated for 7 days. The animals were controlled daily for the health symptomatology using standardized score sheets. During the first two postoperative weeks, the oral mucosa and the teeth were disinfected three times a week using gauzes soaked in a chlorhexidine solution (0.12 %, Perio-Aid Tratamiento®, Dentaid, Barcelona, Spain). Subsequently, a toothbrush and a chlorhexidine gel (0.2 %; Chlorhexidine Bioadhesive Gel, Lacer, Barcelona, Spain) were used three times weekly for plaque control. The dogs were fed a soft-pellet diet for 1 week. The sutures were kept in place during the entire healing time.

### 2.4 Histological analysis

Animals were euthanized 8 weeks after the second phase surgery, using an overdose of ketamine and xylazine. Block sections containing the surgical sites were immediately dissected, rinsed with sterile saline, and fixed in 10 % buffered formalin for 48h. Then, 4 specimens per group were decalcified in 10 % EDTA solution at room temperature. After complete decalcification, the specimens were washed under running tap water, then dehydrated in graded ethanol (50 %, 70 %, 90 %, and 100 %) and embedded in paraffin. Serial mesio-distal sections of 5 mm were cut with LEICA, RM 2245, Germany microtome and prepared for Hematoxylin & Eosin (H&E) and Masson Trichrome (MT) staining according to the manufacturer's protocol. The sections were examined under the light microscope (Olympus America Inc.) and images were captured using a Light Microscope built-in camera (LEICA ICC50 HD Camera system) via image software LAS EZ version 3.0.0.

The others 4 specimens per group were prepared for obtaining undecalcified sections.^4^ The defect area was dissected, and the tissue blocks were fixed in 10 % buffered formalin for 48 h. The specimens were then dehydrated in ascending grades of ethanol, using a dehydration machine (ASP 300S, Leica Biosystems) with agitation and vacuum, and infiltrated and embedded in a clear chemically polymerized methyl methacrylate resin block. Mesiodistal sections of 50 μm thickness were cut from the resin blocks using a precision-cutting machine (Metkon's Micracut150 precision cutter). The sections were then polished with 800-grit silicon carbide paper and finally stained with Stevenel's blue and Van Gieson picrofuchsin. The sections were examined using a light stereomicroscope (BX61; Olympus Corp) equipped with a high-resolution digital camera (E330; Olympus Corp).

### 2.5 Immunohistochemical analysis

The decalcified sections were additionally used for immunohistochemical evaluation. For immunohistochemical examination, alkaline phosphatase-1 (ALP-1) staining was performed. Briefly, 5 μm-tissue sections were deparaffinized, rehydrated, and pre incubated overnight in 1 % magnesium chloride in 100 mm Tris-maleate buffer (pH 9.2). Then, sections were incubated in ALP substrate solution for 2 h at room temperature. After washing with distilled water, the sections were counterstained with Mayer's hematoxylin stain^3^.

### 2.6 Histomorphometric analysis

The undecalcified sections, with visible apical and coronal notches, were used for histomorphometric analysis. The histomorphometric landmarks were identified, and the following histomorphometric measurements were performed, with the aid of an experienced blinded examiner, using ImageJ 1.46r software system under the same magnification x100.

- New bone height%: equals new bone height (distance between the apical end of notch B and the most coronal point of newly formed bone) \ total defect height (distance between the apical end of notch A to the apical end of notch B) x100.
- The surface area of newly formed bone%: equals newly formed bone area\ total defect area (area measured between the apical end of notch A to the apical end of notch B) x100.
- Surface area of newly formed PDL. Equals surface area of the PDL region extended from the apical end of notch B to the most coronal extension of newly formed bone.

### 2.7 Statistical analysis

Statistical analyses were performed using Statistical Package for Social Sciences (IBM SPSS Statistics version 26). Numerical variables were expressed by mean, standard deviation, and range. Nominal variables were represented by frequency and percentage. P value < 0.05 (∗) was considered a significant difference & P-value <0.001 (∗∗) was considered a highly significant difference.

The tests used in this analysis:

1 The Shapiro-Wilk test was used to examine the normality of the data.
2 For parametric variables:
  • The independent *t*-test was used to compare differences between the studied groups.

### 2.8 Ultrastructural analysis

Scanning electron microscopy (SEM) was used to analyze the composite scaffold's shape and structure. In brief, the composite scaffold specimens were secured to the platform after being cut into thin parts and secured firmly onto the platform. Following platinum-palladium sputter coating, the sections were analyzed using a Field Emission Scanning Electron Microscope (FESEM, Quattro S, Thermo Scientific). Mercury porosimetry was used to evaluate the average pore size and distribution. Radiograph diffraction analysis (RDX) was used to determine the elemental surface composition.

## 3 Results

### 3.1 Histological results

**Fig. 2:**
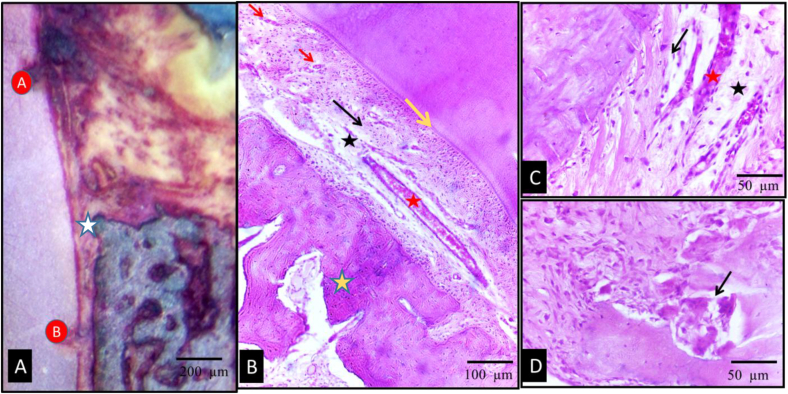
Photomicrographs of the control group showing undecalcified (A) and H&E stained (B–D) sections. (A) The level of the new bone alveolar crest (white asterisk) was almost at the midway between notch A and B. (B&C) showed new bone (yellow asterisk), new cementum (yellow arrow) and new PDL fibers that were mostly vertically oriented (black arrows), increased ERMs clusters (red arrows), wide interstitial spaces (black asterisk) and marked hyperemia (red asterisk) were presented (D) photomicrograph showed osteoclasts (arrow) along the alveolar bone. (original magnificationAx100, Bx200, C&Dx400). (For interpretation of the references to colour in this figure legend, the reader is referred to the Web version of this article.)

**Fig. 3:**
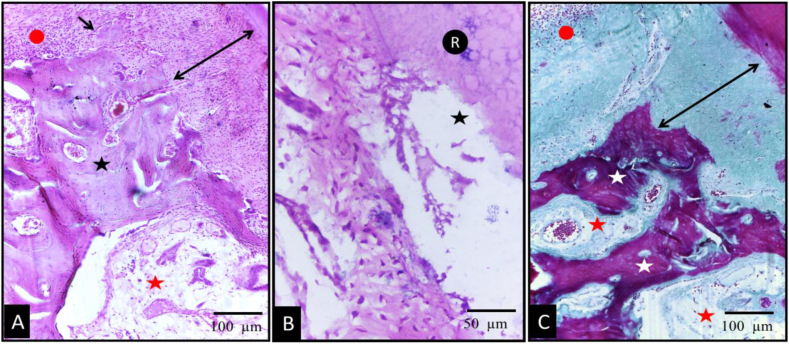
Photomicrographs of control group showing H&E (A&B) and Masson Trichrome (MT) (C) stained sections. (A) Showed newly formed bone (black asterisk) with wide marrow spaces (red asterisk) and few newly formed bony spicules (arrow). (B) One specimen showed advanced stage of root (R) resorption (asterisk). (C) MT stained section showed inflammatory infiltration (red circles) thin new bone trabeculae (white asterisks) separated by wide marrow spaces (red asterisks). Double sided arrow: wide PDL space. (original magnification: A&Cx200, Bx400). (For interpretation of the references to colour in this figure legend, the reader is referred to the Web version of this article.)

Light microscopic examination of the control group, using undecalcified H&E and MT-stained sections, revealed the following findings: The level of the newly formed alveolar bone crest was approximately midway between notches A and B. New cementum and PDL fiber formation were observed. However, complete regeneration of connective tissue attachment, including Sharpey's fibers inserting into new cementum on one side and new bone on the other, was not detected. Instead, the connective tissue fibers were vertically oriented and aligned parallel to the root surface. The quantity of fibers in the newly formed PDL was significantly reduced, and there was a noticeable increase in Epithelial Cell Rests of Malassez (ERM) clusters, large interstitial spaces, and hyperemia (Fig. 2). The PDL space in most regions was predominantly filled with inflammatory cells, with a low density of osteoblasts and fibroblasts. Newly formed bone trabeculae were thin surrounding large marrow spaces. Despite the presence of numerous osteoclasts along the alveolar bone, newly formed bony spicules were also evident. One specimen displayed an advanced stage of root resorption, and a few others showed the gingiva primarily occupied by inflammatory infiltration (Fig. 3).

**Fig. 4:**
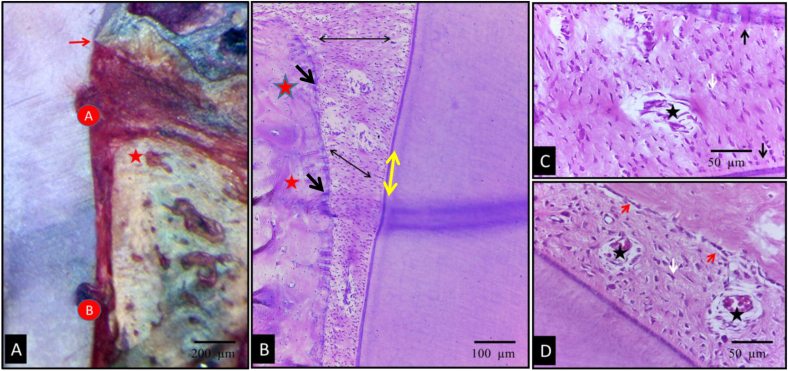
Photomicrographs of the test group showing undecalcified (A) and H&E stained (B–D) sections. (A) The new bone alveolar crest level (red asterisk) was nearer to the CEJ (arrow) and notch A when compared to the control group. (B,C&D); thick well-ordered PDL fibers (double sided arrows) was seen with well-defined Sharpey's fibers (black arrows) inserting into newly formed bone (red asterisk) and cementum (yellow arrows). Newly formed bone was covered by a well-defined osteoblast layer (red arrows), numerous elongated fibroblasts (white arrows), well defined narrow interstitial tissue spaces (black asterisks). (original magnification Ax100, Bx200, C&Dx400). (For interpretation of the references to colour in this figure legend, the reader is referred to the Web version of this article.)

**Fig. 5:**
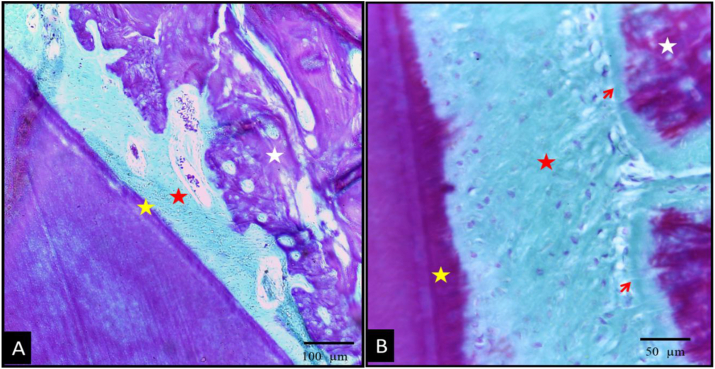
Photomicrographs of the test group (A&B) showing MT stained sections presenting complete regeneration of the periodontal apparatus; new bone (white asterisks) was covered by a well-defined osteoid layer (red arrows), new regenerated PDL (red asterisks); and new cementum formation (yellow asterisks). (original magnification Ax200, Bx400). (For interpretation of the references to colour in this figure legend, the reader is referred to the Web version of this article.)

Upon evaluation of the test group, signs of periodontal regeneration and functional connective tissue attachment were observed. These included well-organized, thick periodontal ligament (PDL) fibers, with Sharpey's fibers clearly inserted into newly formed bone and opposing cementum. Significant bone formation was evident, with new bone covered by a well-defined osteoid layer, lined by a distinct osteoblast layer. The alveolar crest level in the test group was closer to the cementoenamel junction (CEJ) and notch A, compared to the control group (Fig. 4). The inflammatory response observed in group I was notably reduced (see Fig. 5).

MT stained sections presenting complete regeneration of the periodontal apparatus as new bone formation covered by a well-defined osteoid layer new regenerated PDL and new cementum formation group (Fig. 4).

**Fig. 6:**
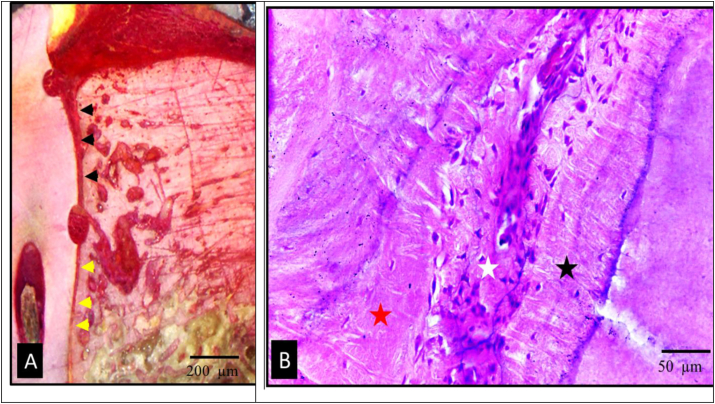
Photomicrographs of the test group. Undecalcified (A) and H&E stained (B) sections showed obvious PDL thinning apical to the defect area. Photomicrograph (A) presented the difference between PDL thickness in the defect area (black arrows) and apical to the defect area (yellow arrows). Photomicrograph (B) showed continuous deposition of bone (red asterisk) and cementum (black asterisk) with marked PDL thinning (white asterisk). (original magnification Ax100, Bx400). (For interpretation of the references to colour in this figure legend, the reader is referred to the Web version of this article.)

However, it is important to note that in three specimens, despite showing significant periodontal regeneration in the defect area, the PDL more apically exhibited a marked reduction in thickness, with a noticeable thickening of the surrounding bone and cementum (Fig. 6).

### 3.2 Immunohistochemical results

**Fig. 7:**
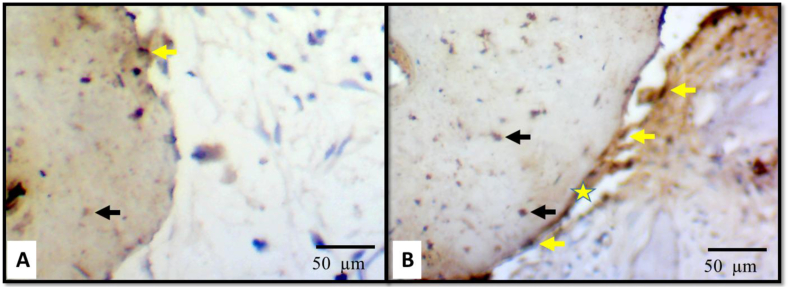
Photomicrographs of control group (A) and test group (B) showing ALP activity. It was seen in relation to the osteoid layer (star), osteoblasts (yellow arrows) and increased newly embedded osteocytes (black arrows). Note that the intensity of ALP expression was more obvious in group II (B) than group I (A). (Immunohistochemical stain; ALP. original magnification x400). (For interpretation of the references to colour in this figure legend, the reader is referred to the Web version of this article.)

ALP activity was observed in both groups, primarily associated with the osteoid layer, osteoblasts, and a few newly embedded osteocytes. The intensity of ALP expression was more pronounced in test group compared to control group (Fig. 7).

### 3.3 Histomorphometric results

**Fig. 8:**
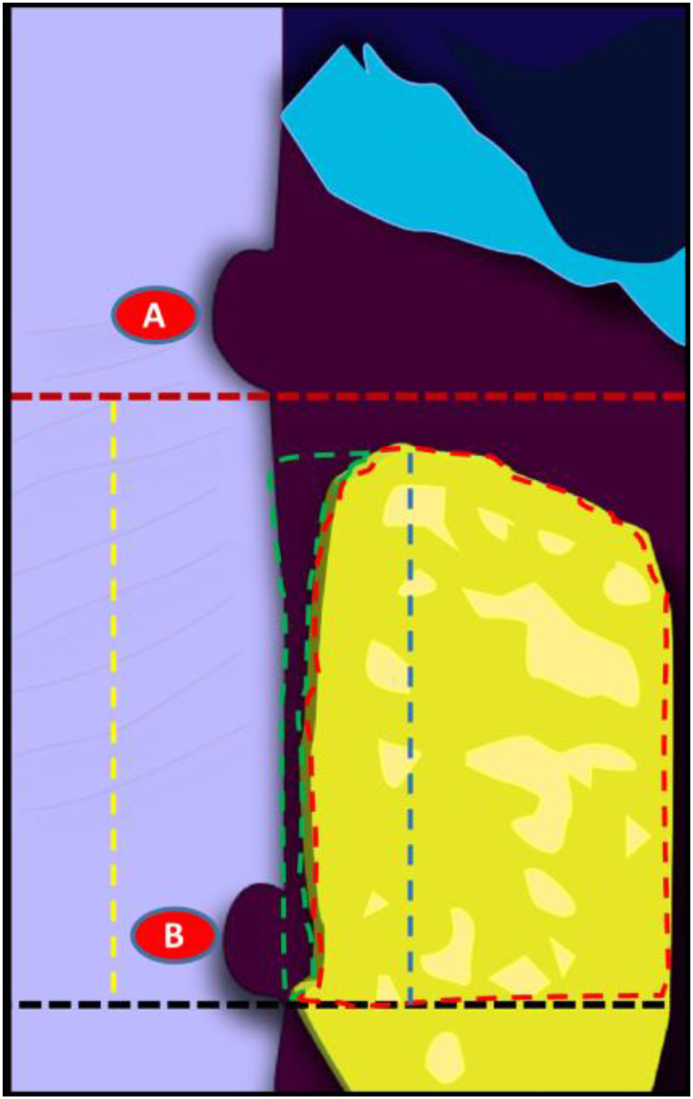
A diagram showing histomorphometric measures. Red dashed line: apical end of notch A, black dashed line: apical end of notch B, yellow dashed line: total defect height, blue dashed line: height of newly formed bone, red dashed area: surface area of newly formed bone, green dashed area: surface area of newly formed PDL. (For interpretation of the references to colour in this figure legend, the reader is referred to the Web version of this article.)

The histomorphometric measures are presented by a diagram showing. Red dashed line: apical end of notch A, black dashed line: apical end of notch B, yellow dashed line: total defect height, blue dashed line: height of newly formed bone, red dashed area: surface area of newly formed bone, green dashed area: surface area of newly formed PDL (Fig. 8).

The histomorphometric data were presented as mean, standard deviation, and range. An independent *t*-test was employed to compare the differences between the studied groups as follows:

- The normality of the data was assessed using the Shapiro-Wilk test, which indicated that the data followed a normal distribution, as shown in Table 1.

**Table 1:** showed normal distribution of the data of bone height, bone surface area and PDL surface area.

Normality test		
Variable	Group 1	Group 2
	Shapiro-Wilk (p-value)	Shapiro-Wilk (p-value)
Bone height %	0.951 (0.745)	0.998 (0.998)
Bone surface area %	0.935 (0.628)	0.859 (0.223)
PDL surface area %	0.997 (0.998)	0.895 (0.380)
- **Percentage of new bone height:**

**Fig. 9:**
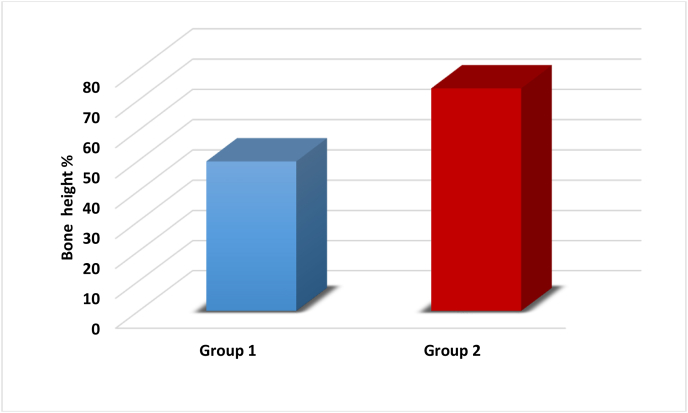
Showing the statistical analysis of Bone Height % of the two studied groups.

The independent *t*-test revealed a highly significant difference between the groups, with a p-value of 0.000∗∗ (Table 2, Fig. 9).

- **Percentage of new bone surface area:**

**Table 2:** P-value< 0.05 (∗) = significant difference, P-value< 0.001 (∗∗) = highly significant difference.

parameter	Bone height %			
	Mean ± S.D	Range	t	p-value
Group 1	49.67 ± 4.50	43.95–55.17	7.606	0.000∗∗
Group 2	73.79 ± 5.48	66.26–80.77

**Fig. 10:**
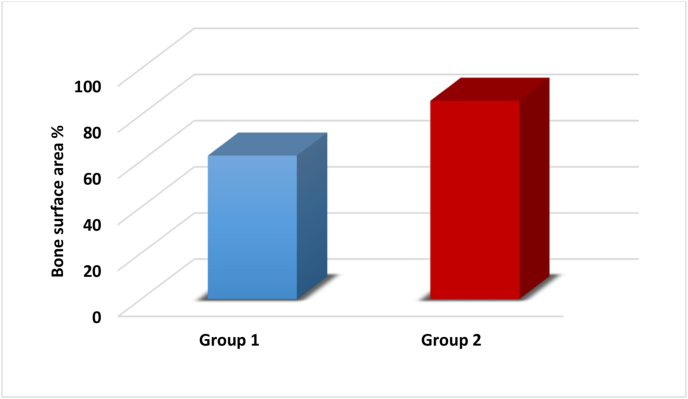
Showing the statistical analysis of Bone surface area % of the two studied groups.

The independent *t*-test demonstrated a highly significant difference, with a p-value of 0.000∗∗ (Table 3, Fig. 10).

- **Surface area of newly formed PDL:**

**Table 3:** P-value< 0.05 (∗) = significant difference, P-value< 0.001 (∗∗) = highly significant difference.

parameter	Bone surface area %			
	Mean ± S.D	Range	t	p-value
Group 1	62.32 ± 2.81	59.17–65.71	8.468	0.000∗∗
Group 2	86.09 ± 5.61	76.93–90.83

**Fig. 11:**
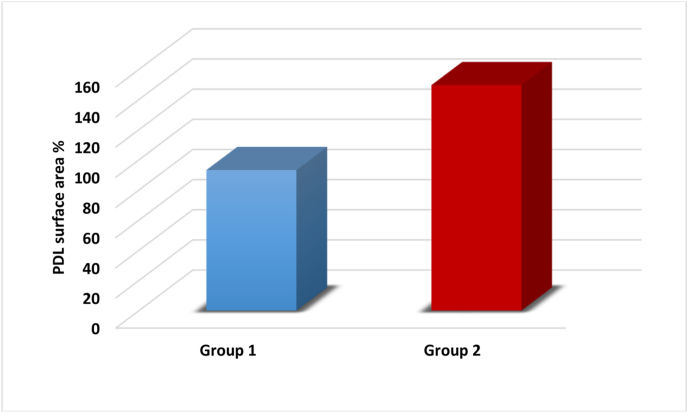
Showing the statistical analysis of PDL surface area % of the two studied groups.

The independent *t*-test revealed a highly significant difference, with a p-value of 0.000∗∗ (Table 4, Fig. 11).

**Table 4:** P-value< 0.05 (∗) = significant difference, P-value< 0.001 (∗∗) = highly significant difference.

Parameter	PDL surface area			
	Mean ± S.D	Range	t	p-value
Group 1	93.24 ± 2.69	89.77–96.97	34.141	0.000∗∗
Group 2	149.59 ± 2.53	145.46–151.97

### 3.4 Ultrastructural results

Scanning electron microscopy (SEM) was used to analyze the composite scaffold's shape and structure. Mercury porosimetry was used to evaluate the average pore size and distribution. Radiograph diffraction analysis (RDX) was used to determine the elemental surface composition. Overall, these SEM observations provide vital information about the structural and morphological characteristics of β-TCP, relevant for its application in biomedical fields such as bone regeneration and tissue engineering.

**Fig. 12:**
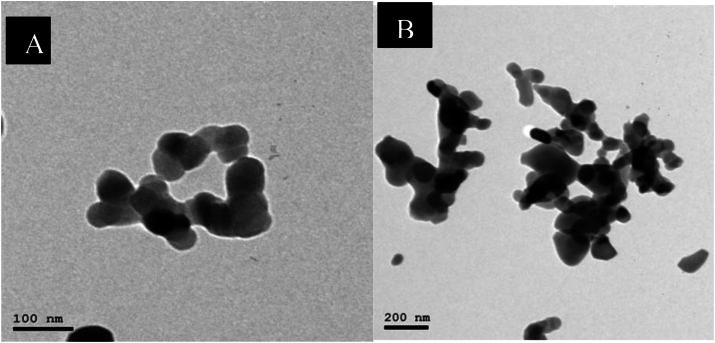
(A) Showing the SEM images showing the spherical shape, size and crystalline structures of the prepared β-TCP Particles Morphology (B) showing evidence of particle aggregation or clustering.

**Fig. 13:**
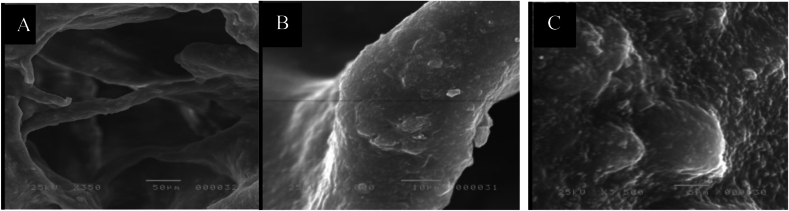
(A): Shows the SEM images of the prepared CMs (GM)-β-TCP/gelatin composite scaffold illustrating the interconnected pores within the porous structure of the scaffold. (B): High-resolution images revealing the surface roughness of the β-TCP/gelatin composite particles (C) Identified gentamicin-loaded chitosan microspheres embedded within the scaffold matrix, illustrating their shape and uniform distribution.

The SEM images showing the spherical shape, size and crystalline structures of the prepared β-TCP Particles Morphology (Fig. 12A): while other SEM images showing evidence of particle aggregation or clustering (Fig. 12B):

The SEM images of the prepared CMs (GM)-β-TCP/gelatin composite scaffold illustrate the interconnected pores within the porous structure of the scaffold (Fig. 13A) A Higher resolution images revealing the surface roughness of the β-TCP/gelatin composite particles (Fig. 13B) The SEM presented identified gentamicin-loaded chitosan microspheres embedded within the scaffold matrix, illustrating their shape and uniform distribution (Fig. 13C).

**Fig. 14:**
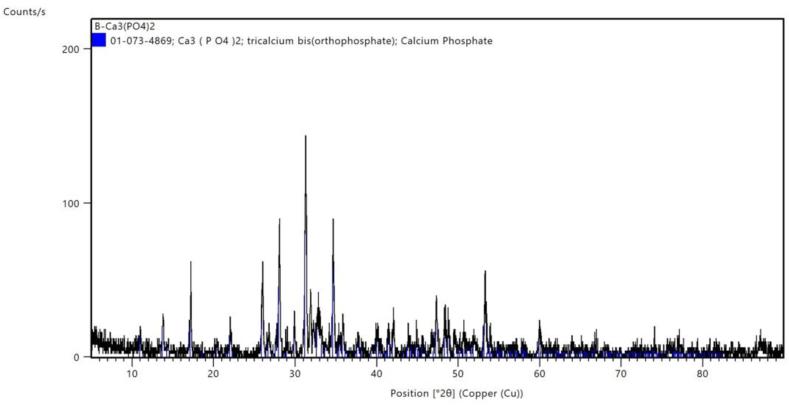
The X-ray diffraction (XRD) pattern of the prepared β-TCP: It displays sharp peaks corresponding to the crystalline phases of β-TCP. The presence of well-defined peaks indicates good crystallinity. Characteristic Peaks: Specific 2θ angles where peaks appear are indicative of β-TCP planes of the crystalline structure.

The X-ray diffraction (XRD) pattern of prepared β-tricalcium phosphate (β-TCP) typically exhibits sharp peaks corresponding to the crystalline phases of β-TCP. In addition to well-defined peaks indicates good crystallinity. The XRD also presented characteristic peaks: Specific 2θ angles where peaks appear are indicative of β-TCP planes of the crystalline structure (Fig. 14).

## 4 Discussion

One the major strengths in the current research study concerned with periodontal regeneration using a novel scaffold by using a canine model, which closely mimics clinical cases in humans. The Combination of histomorphometric, and immunohistochemical assessment ensured a valuable evaluation of periodontal regeneration. These results prove the potential of this biomaterial as a promising approach for periodontal therapy. Importantly, the combination of histological, histomorphometric, and immunohistochemical analyses strengthening the validity of the results that showed enhanced new bone, cementum, and periodontal ligament formation compared to control groups, confirming the confirming the scaffold's effectiveness in promoting periodontal regeneration and highlighting the need for further research to optimize its application in clinical settings.

This study examined the effectiveness of gentamicin-loaded chitosan microspheres combined with β-tricalcium phosphate (β-TCP)/gelatin composite scaffolds for periodontal regeneration in dogs with two-wall intra-bony defect. For histological examination, both decalcified H&E and MT-stained sections, along with undecalcified sections, were prepared to conduct a thorough histological analysis. This included the evaluation of bone, PDL, and cementum conditions, assessing new tissue formation or tissue destruction, as well as the presence of any inflammatory reaction. Additionally, ALP-1 immunohistochemical staining was performed to assess osteoblastic activity and bone formation.

The results show that the experimental group significantly outperformed the control group in terms of periodontal ligament, cementum, and bone regeneration, as well as new tissue production and regeneration findings (p < 0.05).

When interpreting the histological results, the control group demonstrated moderate regeneration, with the newly formed alveolar bone crest positioned roughly midway between notches A and B. The newly formed PDL exhibited a significant reduction in the amount of fibers, which were vertically oriented, resulting in the absence of a functional connective tissue attachment. These findings are consistent with the observations of Shirakata et al., 2021 ^23^ who reported that in the test group where spontaneous bone formation occurred to a limited extent, and the new PDL collagen fibers were either parallel to or detached from the root surface. The modest improvement observed in the defect area may be attributed to the animal's self-healing capacity, as it was not immunologically compromised and did not suffer from any systemic conditions that could impair its healing ability Percie et al., 2020.^19^

The increased ERM clusters, wide PDL space, and hyperemia are common indicators typically observed in cases of periodontal inflammation^8^ The bone and root resorption, along with the low density of osteoblasts observed in this study, could be attributed to the inflammatory mediators, such as TNFα and IL-1β, which are released during inflammation and sensed by bone cells. In response, these cells alter their physiology and function, leading to the activation of bone resorption and/or impairment of bone formation Helal et al., 2019.^5^ These findings were further supported by the immunohistochemical results in the present study, which showed mild ALP activity, indicating limited osteoblastic activity.

Evaluation of the test group revealed clear signs of periodontal regeneration, including thick, well-organized PDL fibers, new cementum, and significant new bone formation, which was lined by a distinct, well-defined osteoblastic layer. These findings were further supported by the strong ALP immunohistochemical reactivity observed in the test group, indicating robust osteoblastic activity.

The histological findings were corroborated by histomorphometric measurements, including new bone height, surface area, and new PDL surface area. A highly significant difference was observed between Group I and Group II across all histomorphometric parameters, with Group II showing superior results. This indicates that the β-tricalcium phosphate/gelatin composite scaffolds, when combined with gentamycin-loaded chitosan microspheres, have an exceptional ability to enhance periodontal regeneration. At the defect locations treated with β-TCP/Gelatin Scaffolds with Gentamicin-Loaded Chitosan, histological analyses showed better periodontal regeneration, new bone production, and greater cellular infiltration. These results highlight the potential of this novel scaffold technology to address the crucial problem of infection control in periodontal therapy in addition to promoting periodontium regeneration.

However, three specimens from the test group exhibited a significant reduction in the thickness of the PDL apical to the defect area, along with a noticeable thickening of the surrounding bone and cementum. These observations suggest the potential for future ankylosis. Similarly, a previous study by Shirakata et al., 2022 ^16^ reported ankylosis in 1 out of 9 specimens treated with rhFGF-2/β-TCP, compared to rhFGF-2 alone, in a one-wall intra-bony defect model in dogs after an 8-week healing period. Also, Lee et al., 2012.,^24^ observed ankylosis in one site of a one-wall, critical-size, intrabony periodontal defect in dogs, implanted with rhGDF-5/β-TCP, following an 8-week follow-up. In contrast, Neamat et al., 2009^25^ found that β-tricalcium phosphate promoted cell proliferation, osteogenesis, and ideal bone regeneration in a dog intrabony defect model after a 3-month follow-up. The discrepancies in histological outcomes across these studies may be attributed to differences in treatment protocols, defect characteristics, and follow-up durations.

Scanning electron microscopy (SEM) was used to analyze the scaffold's shape and structure, the sections were analyzed using a Field Emission Scanning Electron Microscope.

Ultrastructural results using a scanning electron microscope (SEM) for β-tricalcium phosphate (β-TCP) included some observations in the SEM images, the spherical shape, size and crystalline structures of the prepared β-TCP particles morphology showing evidence of particle aggregation or clustering and the material's porosity within the scaffold which eventually can affect the mechanical properties and biological performance. as reported by Zimmerer et al., 2017 in their research study^26^^.^

High-resolution images of the SEM images of the prepared CMs (GM)-β-TCP/gelatin scaffold illustrated interconnected pores within the porous structure of the scaffold facilitate that facilitate cell migration, vascularization and nutrient transport and cell infiltration indicating cellular behavior and biocompatibility Liu et al., 2022.^14^

Other SEM ultrastructural images revealing the surface roughness of the β-TCP/Gelatin Scaffolds with Gentamicin-Loaded Chitosan particles, surface Coating or or bioactive layer formation on the scaffold surface, suggest the initial stages of bone regeneration and integration. Moreover, exhibit the rough texture exhibited by the SEM images of the examined scaffold indicates the potential effect to enhance bioactivity and cell attachment biocompatibility and effectiveness in promoting periodontal regeneration. Poh et al., 2016.^27^

SEM images identified gentamicin-loaded chitosan microspheres embedded within the scaffold matrix, illustrating their shape and uniform distribution and confirmed the presence of these layers and their integration with the surface of β-TCP/Gelatin Scaffolds with Gentamicin-Loaded Chitosan and help assessment of the uniformity and dispersion of gentamicin-loaded chitosan microspheres within the composite scaffold, which can influence drug release and antibacterial properties.

Radiograph diffraction analysis (RDX) was used to determine the elemental surface composition. In our study, XRD pattern of the prepared β-TCP displays sharp peaks corresponding to the crystalline phases of β-TCP and Characteristic Peaks indicative of β-TCP planes of the crystalline structure. The presence of well-defined peaks indicates good crystallinity and purity of prepared β-TCP or absence of impurities of the synthesized material.

Important details on the morphological and structural properties of β-TCP were revealed by the SEM studies, which are pertinent to its use in biological domains like tissue engineering and bone regeneration. Additionally, if there are several phases present, the intensity of the peaks can be utilized for quantitative phase analysis, which aids in identifying the sample's composition. All things considered, the SEM pictures and the XRD pattern offer vital information about the β-TCP scaffolds' structural, crystallographic, and functional traits as well as their effectiveness in periodontal regeneration, all of which are critical for their use in biomedical domains.

These outcomes can be attributed to the combined actions of the therapeutic agents used in this group. First, β-TCP, a key inorganic component of natural bone, is known for its exceptional osteoconductive and osteoinductive properties Thompson, K. et al., 2019.^9^ Second, gelatin, a natural substance containing peptides and proteins with the Arg-Gly-Asp (RGD) peptide sequence, provides a crucial molecular binding domain for interactions with host cells, promoting cell attachment and tissue reconstruction Atia. et al. 2022^10^^.^ Third, chitosan has demonstrated cell growth activity, serving as a conducive medium for cell adhesion and proliferation, which enhances wound healing and accelerates tissue regeneration, Singh et al., 2010.^28^ Numerous synergistic mechanisms are responsible for the regeneration benefits seen in this investigation. It is well known that β-TCP has osteoconductive qualities that help osteoblasts migrate and attach as reported by Wang et al., 2020.^29^ In addition to the scaffold's biodegradable nature, the gelatin matrix promotes cell survival by improving nutrient retention and cell adhesion. According to Shi et al., 2024,^30^ the use of gentamicin-loaded chitosan microspheres effectively mitigates bacterial infections that can obstruct recovery by allowing for continuous antibacterial action in addition to regulated antibiotic release. The biological and mechanical difficulties related to periodontal regeneration are addressed by this multifaceted strategy.^31^

Furthermore, it is widely known that chitosan can improve the scaffold's mechanical qualities while also having antibacterial capabilities.^12^ According to research showing that drug-loaded chitosan scaffolds can greatly improve tissue healing, the combination of these materials produces a favorable microenvironment for periodontal regeneration.^10^

The marked diminution of the inflammatory reaction here was explained by the dual action of chitosan and gentamycin. Chitosan has proved to attain strong bactericidal and antibacterial activities.^12^ It can inhibit the growth of periodontal pathogens, such as Porphyromonas gingivalis and Aggregatibacter actinomycetemcomitans, and modulate the inflammatory response in human gingival fibroblasts. It also exerts an astonishing anti-inflammatory action through reducing IL-1β–stimulated PGE_2_ production by PDL fibroblasts.^13^

Similarly, gentamycin demonstrates a potent inhibitory effect against both gram-positive and gram-negative bacteria. It has been shown to effectively kill *Staphylococcus aureus*, a bacterium commonly associated with bone and periodontal infections.^14^

The results of this study support and expand on previous studies on the application of biomaterials for periodontal regeneration. The efficacy of β-TCP/gelatin scaffolds in healing infected bone defects has been shown in earlier investigations, such as those by Liu et al. (2022),^14^ highlighting the significance of scaffold composition in improving regenerative results. The relevance of our strategy in controlling infection while fostering healing is supported by Usui et al., in 2021 emphasis on the mechanisms of alveolar bone degradation in periodontitis, particularly the function of periodontal bacteria.^1^

Furthermore, in their discussion on periodontics' future, Kornman et al. (2017) promoted the use of cutting-edge tools and methods to boost treatment effectiveness.^3^ Since earlier research done by Thompson et al., 2019 showed that antibiotic-loaded scaffolds can successfully prevent infection during the healing process,^9^ our study's use of gentamicin-loaded chitosan microspheres represents a significant improvement in this area. By adding to the increasing amount of data supporting the use of multifunctional scaffolds in periodontal regeneration, this study may improve clinical results. Furthermore, it is widely known that chitosan can improve the scaffold's mechanical qualities while also having antibacterial capabilities.^12^

According to research showing that drug-loaded chitosan scaffolds can greatly improve tissue healing, the combination of these materials produces a favorable microenvironment for periodontal regeneration.^11^

Our discoveries have significant clinical ramifications for both human and veterinary medicine. The improved regeneration results seen with our composite scaffold imply that this method may greatly benefit the management of periodontal disease. In cases of chronic periodontitis, where infection control is crucial, the use of scaffolds loaded with antibiotics may be very advantageous,^32^ the results may open the door for future therapeutic applications in human patients and are consistent with Chapple et al. (2015)'s emphasis on customized treatment modalities in periodontics.^2^

Furthermore, the materials employed in our study must be safe and biocompatible in order to be successfully applied. Chitosan and its derivatives are good candidates for periodontal applications because of their well-established biocompatibility. Additionally, adding gentamicin may improve the entire regenerative process in addition to helping to control infections.^5^

As mentioned by Mohamed Abdelfattah et al., in 2025,^17^ future studies should concentrate on improving the scaffolds' composition and maybe investigating the addition of growth factors or other biopolymers to further boost their capacity for regeneration. Translating these findings into clinical practice will need examining the long-term effectiveness of this strategy in different kinds of soft tissue and bone abnormalities. It is advised to do mechanistic research to investigate the signaling pathways and cellular connections involved in the regeneration of both soft and hard tissues.

Furthermore, as demonstrated in earlier studies by Aboushelib & Shawky, 2017^6^ and Elborolosy et al., 2023,^33^ additional research could look into changes to the scaffold composition or different drug delivery methods that might improve the β-TCP/gelatin scaffolds' osteogenic potential, biocompatibility, antibacterial, and anticancer effects. Investigating this method's potential for different kinds of bone or soft tissue regeneration may also provide insightful information.

In summary, this study advances our understanding of periodontal regeneration by showing how β-TCP/gelatin composite scaffolds loaded with gentamicin-laden chitosan microspheres might enhance the effectiveness of treatment for periodontal disease.

## 5 Limitations

Although the current study shows that the used scaffolds with help regenerative potential and validated and therapeutically applicable framework was established through a canine model; but future studies involving human subjects may help to evaluate the translational potential. The sample size and observation period of 8 weeks were sufficient to identify significant differences between groups; however, larger cohorts and extended follow-up durations could provide enhanced insights into the long-term stability of regenerated tissues. Moreover, the study concentrated on standardized two-wall intrabony defects and a singular antimicrobial agent (gentamicin); subsequent research should explore various defect morphologies, alternative antimicrobials, and combination therapies. Despite of the histomorphometric investigations demonstrated clear regeneration outcomes, but molecular analysis may be beneficial. Although the results of this study set the stage for future developments in periodontal therapies for humans and dogs but future studies should focus on long-term outcomes and potential patient-specific adaptations to maximize the benefits of this innovative treatment. Collectively, these limitations and considerations highlight promising directions for advancing this approach toward clinical application.

## 6 Conclusions

Within the limitations of this study, the novel treatment approach using β-TCP/Gelatin Scaffolds with Gentamicin-Loaded Chitosan improved periodontal regeneration and wound healing in two-wall intra bony defects in dogs and could be considered for clinical applications.

## Recommendations

- Further clinical trials are recommended to investigate the application of novel treatment approach using β-TCP/Gelatin Scaffolds with Gentamicin-Loaded Chitosan for improvement of periodontal regeneration and wound healing in two-wall intra bony defects.
- Further research in this field is necessary to improve regenerative medicine techniques and eventually help patients with periodontal disorders,.

## Authors' contributions

- **Conception and design of study:** Mohamed Hamdy Helal, Aya Anwar Alsherif, Yasmin Hamdy, Malak Yousef Mohamed Shoukheba, Khaled M. Ali and Moustafa Nabil Aboushelib.

- **Acquisition of data:** Mohamed Hamdy Helal, Aya Anwar Alsherif, Yasmin Hamdy, Malak Yousef Mohamed Shoukheba, Khaled M. Ali, Mohamed Yehia Abdelfattah and Moustafa Nabil Aboushelib.

- **Analysis and/or interpretation of data:** Mohamed Hamdy Helal, Aya Anwar Alsherif, Yasmin Hamdy, Malak Yousef Mohamed Shoukheba, Khaled M. Ali, Mohamed Yehia Abdelfattah and Moustafa Nabil Aboushelib.

- **Drafting the manuscript:** Mohamed Hamdy Helal, Aya Anwar Alsherif, Yasmin Hamdy, Malak Yousef Mohamed Shoukheba, Khaled M. Ali, Mohamed Yehia Abdelfattah and Moustafa Nabil Aboushelib.

- **Revising the manuscript critically for important intellectual content:** Mohamed Hamdy Helal, Aya Anwar Alsherif, Yasmin Hamdy, Malak Yousef Mohamed Shoukheba, Khaled M. Ali, Mohamed Yehia Abdelfattah and Moustafa Nabil Aboushelib.and Dr.Lamis Hussein.

## Ethical approval

The Study protocol and methodology was conducted following the ethical principles for medical research involving animals and human subjects, approved by the Research Ethics Committee (REC) of Tanta University specifying conditions and constraints for conducting and publishing studies involving animal models (approval no #R-OMPDR-8-23-5). and followed the ARRIVE guideline.^19^REC is organized and operated according to Enhancing Research Ethics Committees in Egypt, Guidelines for Standard Operating Procedures, Monitor 2006, and Guidelines of the Declaration of Helsinki, International Conference of Harmonization ICH, and United States Codes of Federal Regulations.

## Ethics approval and consent to participate

This research titled: A Novel Approach Utilizing Β-Tricalcium Phosphate/Gelatin Composite Scaffolds Incorporated with Gentamycin- Loaded Chitosan Microspheres for Periodontal Regeneration in Two-Wall Intra Bony Defects in Dogs” was conducted following the ethical principles for medical research involving animals and human subjects, and was approved by The research Ethics Committee (REC) of Tanta University specifying conditions and constraints for conducting and publishing studies involving animal models (approval no #R-OMPDR-8-23-5). and followed the ARRIVE guideline REC is organized and operated according to Enhancing Research Ethics Committees in Egypt, Guidelines for Standard Operating Procedures, Monitor 2006, and Guidelines of the Declaration of Helsinki, International Conference of Harmonization ICH, and United States Codes of Federal Regulations.

## Funding

This research was supported through self-funding by the authors.

## Declaration of competing interest

The authors declare that they have no known competing financial interests or personal relationships that could have appeared to influence the work reported in this paper.

## Data Availability

The row data of this research could be obtained from the authors upon request.

## List of Abbreviations:

REC: Research Ethics Committee
β-TCP/Gelatin: β-tricalcium phosphate/gelatin
H&E: Hematoxylin & Eosin
MT: Masson Trichrome
(XRD) pattern: The X-ray diffraction
PDL: periodontal ligament
CEJ: Cemento enamel junction
SEM: Scanning electron microscopy)
rhFGF-2: Recombinant human fibroblast growth factor‐2
DBBM: Deproteinized bovine bone mineral

## References

[1] Usui M., Onizuka S., Sato T., Kokabu S., Ariyoshi W., Nakashima K. (2021). Mechanism of alveolar bone destruction in periodontitis - periodontal bacteria and inflammation. Japanese Dental Sci Rev.

[2] Chapple I.L., Van der Weijden F., Doerfer C. (2015). Primary prevention of periodontitis: managing gingivitis. J Clin Periodontol.

[3] Kornman K.S., Giannobile W.V., Duff G.W. (2017). Quo vadis: what is the future of periodontics? How will we get there?. Periodontol 2000.

[4] Sakai K., Hashimoto Y., Baba S., Nishiura A., Matsumoto N. (2011). Effects on bone regeneration when collagen model polypeptides are combined with various sizes of alpha-tricalcium phosphate particles. Dent Mater J.

[5] Helal M.H., Hendawy H.D., Gaber R.A., Helal N.R., Aboushelib M.N. (2019). Osteogenesis ability of CAD-CAM biodegradable polylactic acid scaffolds for reconstruction of jaw defects. J Prosthet Dent.

[6] Aboushelib M.N., Shawky R. (2017). Osteogenesis ability of CAD/CAM porous zirconia scaffolds enriched with nano-hydroxyapatite particles. Int J Implant Dent.

[7] Annabi N., Nichol J.W., Zhong X. (2010). Controlling the porosity and microarchitecture of hydrogels for tissue engineering. Tissue Eng Part B.

[8] Wirth P., Grosgogeat B., Lagneau C., Jaffrezic-Renault N., Ponsonnet L. (2008). Biomaterial surface properties modulate in vitro rat calvaria osteoblasts response: roughness and or chemistry?. Mater Sci Eng C.

[9] Thompson K., Petkov S., Zeiter S. (2019). Intraoperative loading of calcium phosphate-coated implants with gentamicin prevents experimental Staphylococcus aureus infection in vivo. PLoS One.

[10] Atia G.A.N., Shalaby H.K., Zehravi M. (2022). Drug-loaded chitosan scaffolds for periodontal tissue regeneration. Polymers.

[11] Su Y., Zhang B., Sun R. (2021). PLGA-based biodegradable microspheres in drug delivery: recent advances in research and application. Drug Deliv.

[12] Rizeq B.R., Younes N.N., Rasool K., Nasrallah G.K. (2019). Synthesis, bioapplications, and toxicity evaluation of chitosan-based nanoparticles. Int J Mol Sci.

[13] Khattak S., Wahid F., Liu L.P. (2019). Applications of cellulose and chitin/chitosan derivatives and composites as antibacterial materials: current state and perspectives. Appl Microbiol Biotechnol.

[14] Liu Y., Zhao Q., Chen C., Wu C., Ma Y. (2022). β-tricalcium phosphate/gelatin composite scaffolds incorporated with gentamycin-loaded chitosan microspheres for infected bone defect treatment. PLoS One.

[15] Habib S., Sadek H., Hasanin M., Bayoumi R. (2021). Antimicrobial activity, physical properties and sealing ability of epoxy resin-based sealer impregnated with green tea extract-chitosan microcapsules: an In-Vitro study. Egypt Dent J.

[16] Shirakata Y., Setoguchi F., Sena K. (2022). Comparison of periodontal wound healing/regeneration by recombinant human fibroblast growth factor-2 combined with β-tricalcium phosphate, carbonate apatite, or deproteinized bovine bone mineral in a canine one-wall intra-bony defect model. J Clin Periodontol.

[17] Abdelfattah M., Dewedar Ola, Abdallah M. (2025). Regenerative potential of concentrated growth factor (cgf) membrane in an induced bone defect model in rabbits: a light microscopic and histomorphometric study. Egypt Dent J.

[18] Hamid A.M.F.A., Abou-Zeid A.W.A., Abdelfattah M.Y.S. (2023). Root coverage surgeries using modified tunneling technique of xenogenic collagen matrix versus autologous connective tissue graft as a treatment of Miller class II gingival recession (RCT study). Beni-Suef Univ J Basic Appl Sci.

[19] Percie du Sert N., Hurst V., Ahluwalia A. (2020). The ARRIVE guidelines 2.0: updated guidelines for reporting animal research. PLoS Biol.

[20] Charan J., Biswas T. (2013). How to calculate sample size for different study designs in medical research?. Indian J Psychol Med.

[21] Ogawa K., Miyaji H., Kato A. (2016). Periodontal tissue engineering by nano beta-tricalcium phosphate scaffold and fibroblast growth factor-2 in one-wall infrabony defects of dogs. J Periodontal Res.

[22] Naraharisetti P.K., Lew M.D., Fu Y.C., Lee D.J., Wang C.H. (2005). Gentamicin-loaded discs and microspheres and their modifications: characterization and in vitro release. J Contr Release : Off J Controlled Release Soc.

[23] Shirakata Y., Sena K., Nakamura T. (2021). Histological evaluation of gingival and intrabony periodontal defects treated with platelet-rich fibrin using different protocols: a canine study. Oral Health Prev Dent.

[24] Lee J.S., Wikesjö U.M., Park J.C. (2012). Maturation of periodontal tissues following implantation of rhGDF-5/β-TCP in one-wall intra-bony defects in dogs: 24-week histological observations. J Clin Periodontol.

[25] Neamat A., Gawish A., Gamal-Eldeen A.M. (2009). beta-Tricalcium phosphate promotes cell proliferation, osteogenesis and bone regeneration in intrabony defects in dogs. Arch Oral Biol.

[26] Zimmerer R.M., Jehn P., Kokemüller H. (2017). In vivo tissue engineered bone versus autologous bone: stability and structure. Int J Oral Maxillofac Surg.

[27] Poh P.S.P., Hutmacher D.W., Holzapfel B.M., Solanki A.K., Stevens M.M., Woodruff M.A. (2016). In vitro and in vivo bone formation potential of surface calcium phosphate-coated polycaprolactone and polycaprolactone/bioactive glass composite scaffolds. Acta Biomater.

[28] Singh D., Singh M., Saraf S., Dixit V.K., Saraf S. (2010). Optimization and characterization of gentamicin loaded chitosan microspheres for effective wound healing. Indian J Pharm Educ Res.

[29] Wang W., Meng Q., Li Q. (2020). Chitosan derivatives and their application in biomedicine. Int J Mol Sci.

[30] Shi S., Lu W., Gu X., Lin Q. (2024). Efficacy of gentamicin-loaded chitosan nanoparticles against *Staphylococcus aureus* internalized in osteoblasts. Microb Drug Resist.

[31] Kim Y., Zharkinbekov Z., Raziyeva K. (2023). Chitosan-based biomaterials for tissue regeneration. Pharmaceutics.

[32] Osorio R., Alfonso-Rodríguez C.A., Osorio E. (2017). Novel potential scaffold for periodontal tissue engineering. Clin Oral Invest.

[33] Elborolosy S.A., Hussein L.A., Mahran H. (2023). Evaluation of the biocompatibility, antibacterial and anticancer effects of a novel nano-structured fe-mn-based biodegradable alloys in-vitro study. Heliyon.

